# Sensitivity of Lumbar Total Joint Replacement Contact Stresses Under Misalignment Conditions—Finite Element Analysis of a Spine Wear Simulator

**DOI:** 10.3390/bioengineering12030229

**Published:** 2025-02-24

**Authors:** Steven M. Kurtz, Steven A. Rundell, Hannah Spece, Ronald V. Yarbrough

**Affiliations:** 1 Gyroid LLC, Haddonfield, NJ 08033, USA; hspece@gyroidllc.com; 2 School of Biomedical Engineering, Science and Health Systems, Drexel University, Philadelphia, PA 19104, USA; 3 Explico, Inc., Novi, MI 48375, USA; steve@explico.com; 4 3Spine, Inc., Chattanooga, TN 37402, USA; ron.yarbrough@3spine.com

**Keywords:** spine, arthroplasty, validation, implant alignment, highly crosslinked polyethylene, wear, contact pressure, von Mises stress, effective strain

## Abstract

A novel total joint replacement (TJR) that treats lumbar spine degeneration was previously assessed under Mode I and Mode IV conditions. In this study, we relied on these previous wear tests to establish a relationship between finite element model (FEM)-based bearing stresses and in vitro wear penetration maps. Our modeling effort addressed the following question of interest: Under reasonably worst-case misaligned conditions, do the lumbar total joint replacement (L-TJR) polyethylene stresses and strains remain below values associated with Mode IV impingement wear tests? The FEM was first formally verified and validated using the risk-informed credibility assessment framework established by ASME V&V 40 and FDA guidance. Then, based on criteria for unreasonable misuse outlined in the surgical technique guide, a parametric analysis of reasonably worst-case misalignment using the validated L-TJR FEM was performed. Reasonable misalignment was created by altering device positioning from the baseline condition in three scenarios: Axial Plane Convergence (20–40°), Axial Plane A-P Offset (0–4 mm), and Coronal Plane Tilt (±20°). We found that, for the scenarios considered, the contact pressures, von Mises stresses, and effective strains of the L-TJR-bearing surfaces remained consistent with Mode I (clean) conditions. Specifically, the mechanical response values fell below those determined under Mode IV (worst-case) boundary conditions, which provided the upper-bound benchmarks for the study (peak contact pressure 83.3 MPa, peak von Mises stress 32.2 MPa, and peak effective strain 42%). The L-TJR was judged to be insensitive to axial and coronal misalignment under the in vitro boundary conditions imposed by the study.

## 1. Introduction

Lumbar total joint replacement (L-TJR) was developed to treat degeneration affecting the anterior, middle, and posterior columns of the lumbar spine [1,2]. Historically, large total joint replacements, such as those for the knee, have adopted an anatomic or functional design paradigm [3]. In this context, the present L-TJR is a functional design that replaces both the facet joints and the intervertebral disc, with the goal of providing physiologic allowances and restrictions to various translational and rotational degrees of freedom.

To achieve its functional goal, the L-TJR consists of bilateral metal-on-polyethylene bearings fabricated from CoCr alloy and Vitamin E-stabilized highly crosslinked polyethylene (VE-HXLPE). Based on published international standards, researchers previously evaluated the L-TJR design under idealized “clean” wear test conditions (Mode I), abrasive wear conditions (Mode III), and worst-case sagittal plane impingement (Mode IV) conditions [4]. In these wear test scenarios, the wear of the L-TJR was comparable to that observed in anterior lumbar total disc replacements (ADRs) [4].

The L-TJR has advanced to clinical trials as part of an Investigational Device Exemption (IDE) study [5]. One goal of the clinical trial is to understand the design performance in terms of clinical outcomes and physical functions (e.g., wear and impingement) and how this performance is affected by patient and clinical factors. However, the complexity of the in vivo environment limits the ability to study these effects independently. Furthermore, because the implantation of the L-TJR is performed using manual instrumented techniques [6], it is unclear how variations in component placement in the coronal and sagittal planes may impact device performance. For the present study, we sought to analyze the sensitivity of the L-TJR’s polyethylene stresses and strains associated with surface damage, including the contact pressure, von Mises stress, and effective or von Mises strain, to placement and positioning using computational modeling. Our approach to analyzing misalignment is based on previous research [7,8,9], which provides the basis for what is considered to be severe misalignment conditions in the sagittal plane associated with impingement for lumbar disc replacement.

The goals of this study were to (1) create, verify, and validate a finite element model (FEM) of the L-TJR under Mode I and Mode IV wear conditions, (2) explore a range of axial and coronal misalignment scenarios using the validated FEM under Mode I wear boundary conditions, and (3) assess the sensitivity of the L-TJR mechanical response (contact pressure, von Mises stress, and effective strain) to misalignment by comparing the results to previous in vitro testing. We hypothesized that the stresses and strains of the polyethylene associated with surface damage under axial and coronal plane misalignment conditions would be less severe than those encountered during Mode IV testing and would be comparable to those associated with Mode I testing.

## 2. Materials and Methods

### 2.1. FE Model Development and Validation

#### 2.1.1. FE Model Geometry

Finite element models of the L-TJR (MOTUS^®^, 3Spine, Chattanooga, TN, USA) were developed based on CAD geometries of the in vitro Mode I and Mode IV wear testing setups [4]. Specifically, the model consisted of bilateral implants, each with a cobalt chrome (CoCr)-backed direct compression-molded VE-HXLPE cranial component and a CoCr caudal component. Further details of the implant design have been described previously [2,4].

The implant components were oriented to replicate the baseline configuration (axial convergence angle 40°, anterior–posterior offset 0 mm, coronal tilt 0°) of the previous Mode I and Mode IV wear tests. The model also consists of two platens (vertebral test blocks) to represent the in vitro wear testing setup described in ISO 18192-11 [10]. Two L-TJR implant sizes (referred to as “long” and “short”) were modeled, consistent with the sizes that were physically tested in previous experiments.

Modules within ANSYS software (ANSYS Inc., Canonsburg, PA, USA, version R15) were used for finite element modeling. The polyethylene and CoCr endplate components were discretized with fully integrated 4-point 10-node tetrahedral elements. Mesh sizes for the polyethylene component were varied in order to create four distinct levels of model discretization. Ultimately, the element size of 0.6 mm was determined to be sufficiently converged (i.e., the point after which peak contact stresses or pressures remained within 1% of each other). An element size of 0.6 mm corresponded to 152,315 nodes and 104,743 elements within each polyethylene component.

#### 2.1.2. Material Properties

The component material properties defined in the FEM were based on information from published literature (Table 1). Specifically, the top and bottom implant endplates were treated as linear elastic materials, with a modulus of elasticity of 210 GPa and a Poisson’s ratio of 0.3 [11]. The polymer component was based on GUR 1020-E 80 kGy VE-HXLPE and represented using an elastic-plastic material model [12]. Due to their higher elastic modulus relative to polyethylene, the CoCr components of the L-TJR design are effectively rigid with respect to the polyethylene component. Since the deformable polyethylene was mechanically isolated between two effectively infinitely stiff (non-deforming) metallic components, the resulting mechanical response of the polyethylene is insensitive to the details of how the load is delivered through the vertebral test blocks that comprise the wear simulator fixture. They were, therefore, modeled as rigid. A similar approach has been used for decades by Bartel et al. to model metal-backed contact in UHMWPE for total joint replacement [13,14,15,16], which treats the foundation as rigid.

#### 2.1.3. Wear Test Boundary Conditions

**Figure 1:**
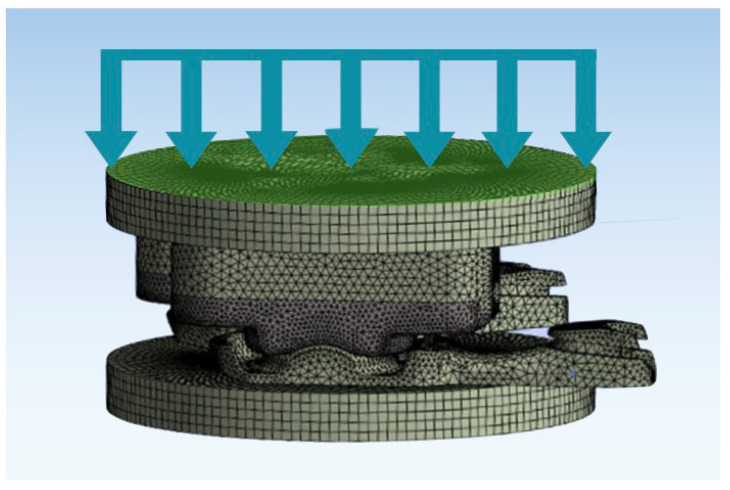
Image depicting the loading conditions of the validated FEM of the L-TJR and in vitro wear simulator. Uniform loading was applied across the superior surface (green surface).

In general, the model was configured such that the nodes on the inferior-most surface were constrained in all six degrees of freedom. Rotational displacements were applied to the superior-most surface by making the nodes on that surface a rigid body. Axial loading was applied in a distributed and uniform fashion to the superior-most surface and always directed in the inferior direction (orthogonal to the inferior-most surface) (Figure 1).

#### 2.1.4. Mode I Wear Duty Cycle

The boundary conditions for Mode I standard wear testing were guided by ISO 18192-1 and ASTM F2423 [10,17]. Specifically, rotations in all three rotational degrees of freedom were prescribed along with axial loading (±4.5° flexion-extension, ±2° lateral bending, ±2° rotation, and 600–2000 N compressive axial load). All translational degrees of freedom were not constrained. Due to the explicit nature of the analysis, the initial condition of the duty cycle was ramped up between an arbitrary time period of 0 to 0.025 s. From there, additional increments of 0.025 s corresponded to 25% of the duty cycle. This shortened time was selected for efficiency purposes. Analyses were performed in such a way as to keep the kinetic energy at zero and limit mass scaling to components that do not move (bottom endplate).

#### 2.1.5. Mode IV Wear Duty Cycle

For the Mode IV unintended wear/impingement analysis, the setup and boundary conditions followed ASTM F3295-18 [18]. For the impingement analysis, an axial force of 1200 N was applied to the reference point on the top block surface, followed by angular extension until posterior contact between the device components (~8°), continuing approximately 2° past initial posterior contact, after which an axial rotation of ±2° was implemented.

#### 2.1.6. Contact Modeling

Contact was defined on the bearing surfaces and at points of potential impingement to accurately simulate physical interactions within the device. LS-Dyna’s (vR15, ANSYS Inc., Canonsburg, PA, USA) “Automatic_Surface_to_Surface” penalty-based algorithm was used, in part for its ability to adapt to changes in the geometry as the simulation progressed. The default penalty factor of 0.1 was used. A uniform coefficient of friction of 0.05 was applied across all contacting interfaces to model the resistance to sliding (static and kinetic) [19,20].

#### 2.1.7. FEM Verification and Validation

We relied on previous Mode I and Mode IV physical wear tests of the MOTUS device to establish a relationship between finite element model (FEM)-based bearing stresses and in vitro wear penetration maps. The FEM was formally verified and validated using the risk-informed credibility assessment framework established by ASME V&V 40-2018 [21] and FDA guidance [22,23]. Briefly, this approach involved formally defining a question of interest (QOI) for the modeling effort and its context of use (COU). A risk analysis was then undertaken to guide the level of rigor that was required for the verification and validation (V&V) activities to establish the credibility of the model. Because the FDA’s guidance for establishing and documenting the credibility of FEMs in accordance with V&V 40 is a relatively new development, examples of such validation efforts are limited in the published literature for spinal implants [24]. We provide in Supplementary Material S1 details on our credibility planning and execution efforts to guide future FEA practitioners and increase familiarity with the risk analysis methodology currently required for regulatory submissions [22,23].

### 2.2. Parametric Analysis and Sensitivity Analysis of Reasonable Axial and Coronal Plane Misalignment

Having established the credibility of the FEM, we turned next to the analysis of axial and coronal plane misalignment. Based on the criteria for unreasonable misuse outlined in the surgical technique guide, a parametric analysis of reasonably worst-case misalignment using the validated L-TJR FEM was performed in the study. Reasonable misalignment was created by altering L-TJR component positioning from the baseline condition for the following three independent misalignment scenarios:

**Axial Plane Convergence Angle**—baseline for Mode I testing was 40° based on reasonable worst-case total convergence between the two components. We therefore parametrically varied the total convergence between 20° and 40°. Less than 20° total convergence (i.e., 10° from the mid-line for each component) is considered unreasonable misuse based on the surgical technique guide and, hence, was beyond the scope of the present study.

**Axial Plane A-P Offset**—baseline for Mode I testing was 0 mm. We parametrically varied the offset between 0 and 4 mm. Greater than 4 mm offset between the two components in the axial plane was considered unreasonable misuse.

**Coronal Plane Tilt**—baseline is 0°. We parametrically increased/decreased coronal tilt with the following four levels: −20°, −10°, +10°, and +20°. Greater than ±20° total convergence (i.e., 10° from the mid-line for each component) is considered unreasonable misuse.

**Figure 2:**
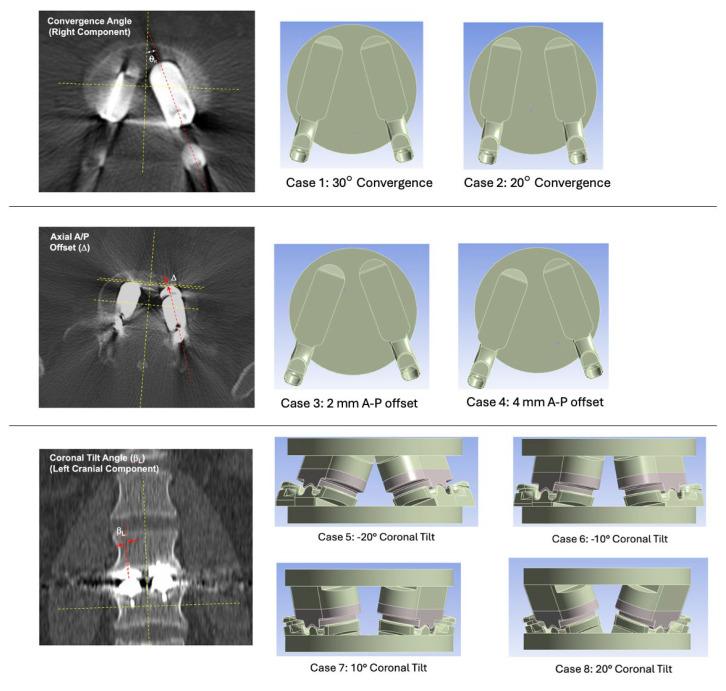
Definitions of convergence angle, axial A/P offset, and coronal tilt angle and images of 3D CAD models illustrating the component positioning of cases 1 through 8.

The following eight simulations of misalignment were conducted, in addition to the Mode I baseline wear testing conditions from the original validation study, as summarized in Table 2 and shown in Figure 2.

The discretization of the L-TJR components in the validated FEM, as well as material properties, boundary conditions, finite element solver parameters, and contact modeling parameters were all scripted parametrically in ANSYS to facilitate the present analysis. Thus, once the orientation of the components was specified, the meshing of the misaligned cases 1 to 8, as well as the additional properties and parameters, were all identical to that of the validated baseline L-TJR FEM.

Resulting contour plots of contact pressure, von Mises stress, and effective strain were collected for each run and at each timestep and compared to values determined during FEM validation (Supplementary Material S1, Validation Goal 2). The Mode IV conditions represent the worst-case loading scenario and, thus, serve as the upper bound benchmark for the stresses and strains associated with surface damage in this study. Therefore, the sensitivity of the design will be judged to be acceptable provided that the contact pressures, von Mises stress, and effective strain determined for each misalignment scenario are less than those determined during simulated Mode IV conditions.

## 3. Results

### 3.1. Sensitivity Analysis of Convergence Angle Misalignment

**Figure 3:**
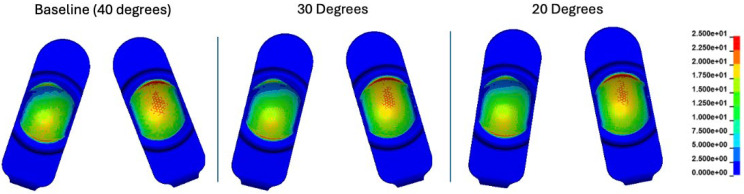
Contour plots representing the superposition of all contact pressures over the Mode I duty cycle for the baseline (40°), 30°, and 20° convergence angle simulations. The contact stress plots are viewed on the L-TJR superior polyethylene components from the bottom looking up. The up direction in the figure corresponds to the anterior direction.

For the baseline model (40° of convergence), the peak contact pressures tended to occur on the periphery of the domed bearing surface. This remained consistent as the convergence angle was decreased to both 30° and 20°. Essentially, areas of contact pressure concentration occurred in the same locations regardless of the convergence angle (Figure 3).

Reducing the convergence angle 20 degrees led to an increase in contact pressure at 35% (28.6 to 38.1 MPa) and 60% (30.1 to 38.2 MPa) of the Mode I duty cycle (Figure S5, Supplementary Material S2). These points in the cycle corresponded to periods of maximum combined axial loading with flexion-extension. During these times, the model experienced a combination of axial loading, flexion-extension, lateral bending, and axial rotation. The peak values were 32.5, 33.7, and 38.2 MPa for the baseline (40°), 30°, and 20° scenarios, respectively. Contour plots of contact pressure at the 35% and 60% timepoints indicated similar contact pressure patterns, qualitatively. Essentially, any divergence caused by the convergence angle at these timepoints was not the result of any non-bearing-surface (Mode IV) contact (Figures S6 and S7).

Peak von Mises stress values throughout the whole Mode I duty cycle were 21.0, 21.1, and 21.3 MPa for the baseline (40°), 30°, and 20° convergence angle simulations, respectively (occurring at 50% of the duty cycle). In general, the pattern and maxima locations of Von Mises stress were generally consistent regardless of convergence angle (Figure S8). Peak effective strain values throughout the whole Mode I duty cycle were 7.1, 7.4, and 7.6% for the baseline (40°), 30°, and 20° convergence angle simulations, respectively. In general, the pattern and maxima locations of effective strain were consistent regardless of the convergence angle (Figure S9). All peak values determined during convergence angle misalignment testing remained lower than those determined by the same FEM during Mode IV impingement boundary conditions.

### 3.2. Sensitivity Analysis of Anterior–Posterior Offset Misalignment

**Figure 4:**
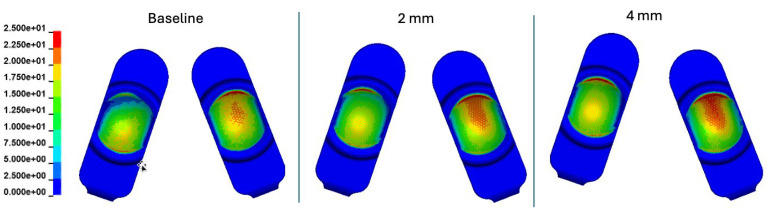
Contour plots representing the superposition of all contact pressures over the Mode I duty cycle for the baseline (0 mm A-P offset), 2 mm A-P offset, and 4 mm A-P offset simulations. The contact stress plots are viewed on the L-TJR superior polyethylene components from the bottom looking up. The up direction in the figure corresponds to the anterior direction.

For the baseline model (zero A-P offset), the peak contact pressures tended to occur on the periphery of the domed bearing surface. This remained generally consistent during A-P offset. However, the anterior aspect of the anterior shifted component (left side on the figures, right side anatomically) exhibited a general increase in contact pressure maxima as can be seen in the contour plots (see Figure 4). Similarly, the anterior aspect of the posteriorly shifted component exhibited generally increased contact pressure maxima when compared to the baseline scenario (Figure 4).

In the early part of the duty cycle (0 to 30%), the A-P offset resulted in a general increase in peak contact pressure (33 to 36%) (Figure S10). Generally, beyond 30%, the peak contact pressures were unaffected by anterior–posterior offset with the small exception of around 60%. The peak contact pressure values were 32.5 MPa, 36.4 MPa, and 37.0 MPa and baseline (0 mm), 2 mm and 4 mm of AP offset, respectively.

Contact pressure contour plots at 10% of the duty cycle depict the location of the increased magnitudes from A-P offset. Specifically, the anterior aspect of the anteriorly shifted component experiences increased contact (Figure S11). Conversely, at 60% of the duty cycle, the anterior aspect of the posteriorly shifted component experiences increased contact pressure (Figure S12). Essentially, these results indicate that A-P offset does not appreciably alter the general patterns of contact pressure but increases pressure magnitude at existing areas of contact.

Peak von Mises stress values throughout the whole Mode I duty cycle were 21.0, 21.6, and 22.5 MPa for the baseline (0 mm), 2 mm A-P offset, and 4 mm A-P offset simulations, respectively. In general, the von Mises stress patterns depicted consistent changes with respect to the contact pressure (Figure S13). Specifically, A-P offset increased existing maxima of von Mises stresses but did not alter the locations of stress maxima. Peak effective strain values throughout the whole Mode I duty cycle were 7.1, 10.5, and 11.6% for the baseline (0 mm), 2 mm A-P offset, and 4 mm A-P offset simulations, respectively (Figure S14).

### 3.3. Coronal Tilt

**Figure 5:**
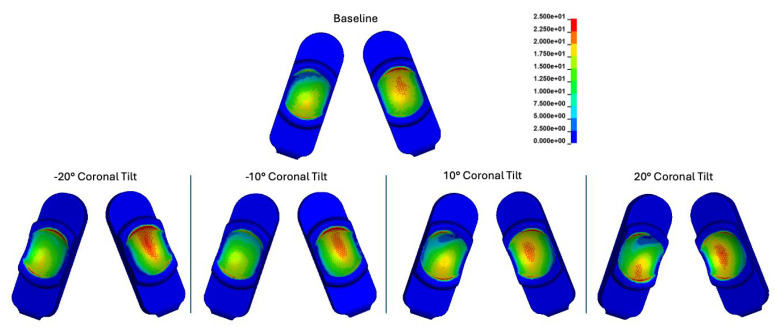
Contour plots representing the superposition of all contact pressures over the Mode I duty cycle for the baseline, −20°, −10°, 10°, and 20° of coronal tilt simulations. The contact stress plots are viewed on the L-TJR superior polyethylene components from the bottom looking up. The up direction in the figure corresponds to the anterior direction.

Overall, coronal tilt had the most significant impact on both the magnitudes and distributions of contact pressure compared to the baseline scenario. In the baseline model (no coronal tilt), peak contact pressures typically occurred on the periphery of the domed bearing surface, a pattern that generally persisted during coronal tilt. However, with positive coronal tilt (i.e., internal rotation of the components), an additional area of contact pressure concentration emerged on the posterior aspect of the left component (right side in the figures), resulting in an apparent reduced contact pressure on the corresponding anterior aspect (Figure 5). Conversely, with negative coronal tilt, the existing area of peak contact pressure generally increased. Ultimately, negative coronal tilt caused the most substantial increase in contact pressure on both sides of the implant.

The peak contact pressures were 32.5 MPa, 40.5 MPa, 33.3 MPa, 35.7 MPa, and 41.6 MPa for the baseline, −20°, −10°, 10°, and 20° of coronal tilt simulations, respectively. For coronal tilt, peak contact pressures relative to baseline occurred at 30% and 60% of the duty cycle (Figure S15). These stresses were generally higher than those experienced during any part of the Mode I duty cycle under baseline implanted conditions. Coronal tilt of the implant generally caused the loading at the bearing surface to shift laterally. This is readily visible in the left-sided component (right side of figures) at 30% and 60% of the duty cycle. The contact pressures in the baseline configuration were primarily centered on the bearing surface and shifted laterally during positive coronal tilt (Figures S16 and S17).

Peak von Mises stress values throughout the whole Mode I duty cycle were 21.0, 23.9, 23.1, 22.6, and 23.8 MPa for the baseline, −20°, −10°, 10°, and 20° of coronal tilt simulations, respectively (Figure S18). Peak effective strain values throughout the whole Mode I duty cycle were 7.1, 18.3, 13.5, 11.4, and 17.1% for the baseline, −20°, −10°, 10°, and 20° of coronal tilt simulations, respectively (Figure S19).

## 4. Discussion

Understanding how device misalignment influences component wear and deformation is essential for characterizing any implantable orthopedic or spinal device. In this study, we created an FEM of a novel L-TJR and validated it against in vitro wear test results. We then used the FEM to assess how the L-TJR polyethylene stresses and strains change under misalignment scenarios and how they compare to values from simulated Mode I and Mode IV in vitro wear testing. We found that for all of the misalignment scenarios considered (Cases 1 to 8), the contact pressures, von Mises stresses, and effective strains fell below the levels associated with Mode IV wear boundary condition performance bench testing. Of the three misalignment types (convergence angle, A-P offset, and coronal tilt), coronal tilt resulted in the greatest change for stresses and strains. Because the stresses and strains associated with surface damage remained consistent with Mode I conditions through a range of worst-case alignment conditions, the L-TJR is judged to be insensitive to reasonable misalignment.

We verified and validated our FEM in accordance with ASME V&V 40. There are currently very few published studies that report using this framework for spinal fusion components [24] and, to date, we have found no such ADR studies for comparison. However, FEM analyses that consider lumbar ADR misalignment have been previously published [25,26,27,28,29,30,31,32,33,34]. Schmidt et al. assessed the effect of sagittal misalignment for a Charité model, specifically displacing the implant by 2 mm both anteriorly and posteriorly [26]. The authors report impacts including range of motion, increased facet joint forces, and lift-off between the device core and endplates (which may lead to dislocation or early component wear). Rohlmann et al. reported similar findings as well as some minor facet joint force effects related to coronal plane alignment (up to 3 mm) [25]. The study by Rundell et al., upon which our present approach is based, additionally reports an increased risk of impingement related to A-P misplacement (as well as implant disk distraction, lordotic angle, and spinal orientation) for a Charité model [8]. Several studies have further demonstrated altered kinematics, facet joint forces, and core stresses due to both sagittal and coronal alignment, with the impact of positioning depending on device type (e.g., mobile or fixed core) and load type (e.g., flexion, extension, bending) [29,30,31,32,33,34].

For the present study, our primary goal was to test the hypothesis that the bearing stresses and strains will be insensitive to reasonable misalignment in the context of Mode IV impingement wear, which is the worst-case test that evaluates conditions beyond reasonable misuse. Overall, the results of our parametric analysis supported our hypothesis, that is, the mechanical response values remained lower than what was determined during simulation under Mode IV conditions (peak contact pressure of 83.3 MPa, peak von Mises stress of 32.2 MPa, and peak effective strain of 42%). Additionally, contact between the superior and inferior components remained confined to the intended spherical bearing surfaces of the design, without evidence of impingement. Based on the literature [16], the contact pressures and internal stresses in the polyethylene indicate that relative risks of wear and surface damage, including pitting, delamination, and fracture associated with misalignment, will be lower relative to the conditions from Mode IV bench testing. Consequently, the existing physical wear test results are sufficient to assess the wear consequences of such misalignment, and the validated FEM will enable the ability to contextualize a wide range of reasonable worst-case misalignment scenarios with wear rates established during physical testing.

Comparison of our work to previous results is limited by the difference in designs: while traditional ADR alignment is judged with respect to the vertebral midline, the bilateral design of the L-TJR introduces the element of alignment between the two components. Additionally, previous studies evaluated devices within lumbar spine models, whereas our work represents a standalone device model under in vitro testing conditions. In the future, our findings will serve as the basis for assessing L-TJR behavior under more complex in vivo loading scenarios.

Our approach was based on historical research [7,8,9] that went into developing what we consider today to be reasonable worst-case misalignment conditions in the sagittal plane for lumbar ADR. This comprehensive body of finite element research was undertaken in response to the well-documented risk of sagittal plane impingement observed in previous motion-preserving spinal implants. Specifically, the development of an impingement model involved not only explant analysis to identify and characterize the potential in vivo damage mode, but also the detailed, validated FEM, conducted with realistic in vivo boundary conditions. Thus, the historical approach towards understanding misalignment is based on an established clinical relevance of sagittal impingement from ADR explants, which were then followed up by simulations with in vivo boundary conditions to reproduce clinically relevant sagittal impingement [35]. These were then used as a basis for developing in vitro sagittal plane impingement tests [18]. We considered anatomical (cadaver) models as an approach to assess wear and deformation of the L-TJR device but ruled them out for several reasons, including availability of representation for the target population, the decreased magnitude of loading that can be exerted on preserved/stored specimens, and limitations with accurate and repeatable misalignment simulation. Overall, our adopted approach lends itself well to translation into more biofidelic boundary conditions, such as those associated with simulated daily activities including standing and bending, and will provide a comprehensive assessment of reasonable worst-case misalignment under a broad set of clinically relevant boundary conditions.

Our analysis has limitations. First, the QOI for our modeling activities is limited to the parametric analysis of reasonable misalignment, not unreasonable misuse or revision scenarios such as subsidence or gross migration. Second, our verification and validation activities undertaken in this report were based on the analysis of a specific LTJR design and relative risk analysis founded on the results of previous Mode I and Mode IV wear tests for that specific design, under the assumption that in future parametric analyses, only the orientation of the components was being perturbed. It is not clear whether this model would be suitable for addressing other QOIs, such as the analysis of scenarios related to revision, which were beyond the scope of our current investigation and would require additional validation activities. Third, the outcomes of our analysis included the bearing stresses and strains in the polyethylene component instead of wear. Previous simulations of PE wear by Maxian and colleagues [36,37] included assessments of bearing stresses and sliding distance into their formulation of Archard’s law for abrasive wear. In the present study, both the implant design factors and duty cycle factors driving the kinematics of the bearing surface remained constant, hence the changes in wear according to Archard’s law would theoretically be driven only by the changes in contact stresses due to misalignment during the duty cycle, when other factors are held constant. Fourth, our boundary conditions were limited to “simulating the simulator”, based on our ability to validate the FEM under these international standard wear test conditions. Thus, certain in vivo conditions were not considered, such as the bone-implant interface, which may have an impact on implant loading. Instead, the constrained inferior portion of the model is “idealized” to allow for a focus on the contact stress and strain at the bearing surface, while subsidence and other bone quality related factors were beyond the scope of our present study. The findings reported here will serve as the basis for assessing L-TJR behavior under more complex in vivo loading scenarios in future research. The application of the model to such alternate scenarios will of course necessitate additional verification and validation activities. Overall, the limitations of the model do not diminish the credibility of the FEM and its ability to rigorously address the QOI for which it was developed. Rather, the limitations identified here are intended to caution the reader about the potential generalization of our findings to other QOIs or COUs without undertaking the appropriate validation activities.

## 5. Conclusions

In this study, we established a validated FEM of a novel lumbar total joint replacement. The L-TJR device stresses and strains associated with surface damage were judged to be insensitive to axial and coronal misalignment under the in vitro boundary conditions imposed by the study in the context of Mode IV (impingement) boundary conditions.

## Figures and Tables

**Table 1:** Material properties used in the finite element model [11,12].

Component	Material	Elastic Modulus (GPa)	Poisson’s Ratio	Yield Strength (MPa)
Endplates	CoCr	210	0.3	N/A
Polymer Bearing Surface	UHMWPE GUR 1020-E 80 kGy	0.662	0.4	11.65

**Table 2:** Summary of axial misalignment conditions for FE models.

Misalignment Condition	Baseline Mode I Scenario	Case 1	Case 2	Case 3	Case 4	Case 5	Case 6	Case 7	Case 8
Axial Convergence Angle (°)	40	30	20	40	40	40	40	40	40
Axial A-P Offset (mm)	0	0	0	2	4	0	0	0	0
Coronal Tilt (°)	0	0	0	0	0	−20	−10	10	20

## Data Availability

The data presented in this study are available on request from the corresponding author.

## Supplementary Materials

The following supporting information can be downloaded at: https://www.mdpi.com/article/10.3390/bioengineering12030229/s1, Table S1: Summary of seven verification and three validation activities performed for the L-TJR FEM in accordance with ASME V&V 40-2018 and FDA guidance; Figure S1: Diagram of L-TJR component damage-scoring regions for validation activities 1 and 2; Figure S2: Contour plots representing the superposition of maximum contact pressures over the duty cycle. Superior component penetration maps based on microCT scans after 10 million cycles of Mode I duty cycles are also shown; Figure S3: Contours of maximum contact pressure across all states; Figure S4: Contours of maximum contact pressure across all states; Figure S5: Plotted peak contact stress values for the Mode I duty cycle for the baseline, 30°, and 20°, convergence angle simulations; Figure S6: Contour plots of contact stress (MPa) at 35% of the Mode I duty cycle for the baseline, 30 degree, and 20 degree convergence angle simulations; Figure S7: Contour plots of contact stress (MPa) at 60% of the Mode I duty cycle for the baseline, 30 degree, and 20 degree convergence angle simulations; Figure S8: Contour plots representing the superposition of all von Mises stresses (MPa) over the duty cycle for the baseline, 30°, and 20° convergence angle simulations; Figure S9: Contour plots representing the superposition of all effective strains over the duty cycle for the baseline, 30°, and 20° convergence angle simulations; Figure S10: Plotted peak contact pressure values for the Mode I duty cycle for the 2 mm A-P offset, and 4 mm A-P offset simulations; Figure S11: Contour plots of contact pressure at 10% of the Mode I duty cycle for the 2 mm A-P offset, and 4 mm A-P offset simulations; Figure S12: Contour plots of contact pressure at 60% of the Mode I duty cycle for the 2 mm A-P offset, and 4 mm A-P offset simulations; Figure S13. Contour plots representing the superposition of all von Mises stresses over the duty cycle for the 2 mm A-P offset, and 4 mm A-P offset simulations; Figure S14: Contour plots representing the superposition of all effective strains over the duty cycle for the 2 mm A-P offset, and 4 mm A-P offset simulations; Figure S15: Plotted peak contact stress values for the Mode I duty cycle for the baseline, −20°, −10°, 10°, and 20° of coronal tilt simulations; Figure S16: Contour plots of contact stress at 30% of the Mode I duty cycle for the baseline, −20 degrees, −10 degrees, 10 degrees, and 20 degrees of coronal tilt simulations; Figure S17: Contour plots of contact stress at 60% of the Mode I duty cycle for the baseline, −20 degrees, −10 degrees, 10 degrees, and 20 degrees of coronal tilt simulations; Figure S18: Contour plots representing the superposition of all von Mises stresses over the duty cycle for the baseline, −20°, −10°, 10°, and 20° of coronal tilt simulations; Figure S19: Contour plots representing the superposition of all effective strains over the duty cycle for the baseline, −20°, −10°, 10°, and 20° of coronal tilt simulations. References [38,39] are cited in the supplementary materials.
