# Supplementary material for: Sensitivity of Lumbar Total Joint Replacement Contact Stresses Under Misalignment Conditions—Finite Element Analysis of a Spine Wear Simulator
Source: Bioengineering (Basel). 2025 Feb 24;12(3):229. doi: 10.3390/bioengineering12030229 (PMC11939812; doi:10.3390/bioengineering12030229)
Supplement: Supplementary file 1 [file bioengineering-12-00229-s001.zip › bioengineering-3377078-supplementary.pdf]

Supplementary Material 1: FEM Validation

The primary QOI for the FEM was the following: Under reasonably worst-case misaligned conditions, do the lumbar total joint replacement (L-TJR) polyethylene stresses and strains remain below values associated with Mode IV impingement wear tests? Previously performed physical wear testing for both Mode I and Mode IV wear modes were used to establish low wear of the L-TJR design, consistent with historical anterior lumbar total disk replacements [4]; the computational model was developed in the present study to evaluate the sensitivity of the L-TJR design.

The COU for the FEM was to assess the impact of worst-case misalignment scenarios on polyethylene wear through quantification of the bearing surface contact pressure (i.e., contact stress), von Mises stress, and von Mises (i.e., effective) strain. The primary goal was to quantify contact pressures associated with Mode I (a standardized test that is considered a best case for alignment and contact) and Mode IV (the worst-case test that evaluates conditions beyond reasonable misuse) wear to determine if contact pressures occurring during misalignment would match or exceed those that occur during a worst-case Mode IV scenario. Consequently, the model was developed as a tool to assess relative risk of the L-TJR device design to misalignment by comparing the stresses and deformations in the model in reasonably worst-case misaligned scenarios with the results from the impingement (Mode IV) and non-impingement (Mode I) simulations.

The model risk assessment considered both the model influence and decision consequence as required by FDA guidance and ASTM V&V 40 [21-23]. The model influence was found to be "low" because the outputs would only make up a minor factor in answering the QOI [4]. The primary data establishing the safety profile to wear included previous physical wear tests for both Mode I and Mode IV wear [4]. The previous wear tests included the presence of misalignment and erred on the side of worst-case scenarios (i.e., maximum device convergence angle and impingement). The FEM in the present study was intended to supplement these findings by evaluating the sensitivity of contact pressures during reasonable misalignment in the coronal and axial planes. The decision consequence was also classified as "low" because the model was used to assess the sensitivity of stresses relative to two physically tested and benchmarked states that were associated with an acceptable assurance of safety. The credibility plan included seven verification and three validation activities (**Table S1**).

Table S1: Summary of seven verification and three validation activities performed for the L-TJR FEM in accordance with ASME V&V 40-2018 [21] and FDA guidance [22,23].

Activity	Outcome
Verification Goal 1: Code verification	Software credibility certified by ANSYS. We performed the suite of verification benchmark tests provided by ANSYS for LS-DYNA. Benchmark testing demonstrated <1.9% maximum error among 34 test parameters evaluated.
Verification Goal 2: Sufficient discretization of finite element mesh	Four levels of mesh discretization (1.0, 0.8, 0.6, and 0.5 mm) were assessed. Stable convergence of the resulting contact pressures was indicated at an element size of 0.6 mm.
Verification Goal 3: Force balancing given the quasi-static nature of the simulation	Results indicated equivalence between the total superior–inferior contact force and the superior–inferior constraint force. These forces

	were also consistent with the prescribed axial loading per the boundary conditions.
Verification Goal 4: Stress pattern symmetry under symmetric loading	Ran analysis with purely linear-elastic material properties and confirmed that instances of asymmetric contact pressures during symmetric applied loading in the duty cycle was a result of the plasticity of the PE material properties.
Verification Goal 5: Quasi-static performance of explicit analysis	Kinetic energies remained at zero for both Mode I and Mode IV simulations, indicating a quasi-static solution during explicit analysis.
Verification Goal 6: User error mitigation	Key inputs (i.e., material properties, load and displacement boundary conditions, contact definitions, and output displacement and stresses) were independently verified.
Verification Goal 7: Confirm the displacement boundary conditions from the simulator are correctly implemented in the FEM.	A video of the simulator applying the gait cycle to the L-TJR was compared with the FEM. A side-by-side comparison of the FEM and simulator kinematics (order and phasing of rotations) was performed to ensure similarity between the FEM and physical tests.
Validation Goal 1: Comparable contact pressures and polyethylene deformations between FEA and wear maps from Mode I in vitro testing	FE peak contact stress was 39 MPa and occurred at or near to 100% of the duty cycle. Cumulative contact stress distributions from FEM and MicroCT wear maps from the wear tests were compared using damage-scoring techniques and were found to agree within 6.2%.
Validation Goal 2: Comparable contact pressures and polyethylene deformations between FEA and wear maps from Mode IV in vitro testing	FE indicated focal wear and deformation on the posterior aspect of the PE component outside of the intended bearing surface, consistent with physical Mode IV wear testing. Cumulative contact stress distributions from FEM and MicroCT wear maps from the impingement wear tests were compared using damage scoring techniques and were found to agree within 6.7%.
Validation Goal 3: Levels of PE component contact pressures consistent with previously reported and validated magnitudes	Maximum Mode I contact pressures were comparable to previously reported values for large-joint PE-bearing surfaces [16,19].

Validation included direct comparison of cumulative contact pressures (representing the superposition of all contact pressures over the duty cycle) during the simulated duty cycle against MicroCT penetration maps from Mode I (Validation Goal 1) and Mode IV (Validation Goal 2). We selected the L-TJR size (15 mm long) for the Mode I study based on its appropriateness with a previously developed and validated FEM of the lumbar spine (Validation Goal 1). For Mode IV analysis (Validation Goal 2), the impingement testing was performed on the thinnest devices (11 mm long and short sizes), and the FEA, therefore, also utilized these sizes.

We compared the simulation results qualitatively and semi-quantitatively in our validations. The foundation for the comparisons was the penetration or wear maps from the physical Mode I and Mode IV wear tests [4]. These penetration maps for the polyethylene components were based on MicroCT analysis of the wear tested components before and after wear testing. The polyethylene components were scanned using a CT 80 (Scanco Medical AG, Switzerland) at approximately 18 mm voxel resolution as further described by Siskey et al. [4].

Semi-quantitative comparisons between the penetration maps and the cumulative contact pressure distributions were made in six regions of the

polyethylene-bearing surface (**Figure S1**). Within each region, the damage was scored from 0 to 3, with damage being defined as greater than 0.2 mm deformation (corresponding to visually differentiable damage) for the penetration maps and (>2.5 MPa) for the contact pressure contour plots. A grade of 0 indicated that damage was not present in that region. Grades of 1, 2, or 3 indicated that damage was present in <10%, 10-50%, or over 50%, respectively, similar to the methodology established by Hood et al. [38]. The total damage score was obtained by the sum of the scores in all six component regions. The left and right components were scored independently. The wear maps from the physical testing and the cumulative contact pressure plots were scored independently. Damage scoring was performed by three of the study co-authors (SMK, SJR, and HS) who met to adjudicate differences in the scoring. The simulation results were judged as reasonably acceptable if the damage scores in the rim and bearing surface compare within 20% to the penetration maps.

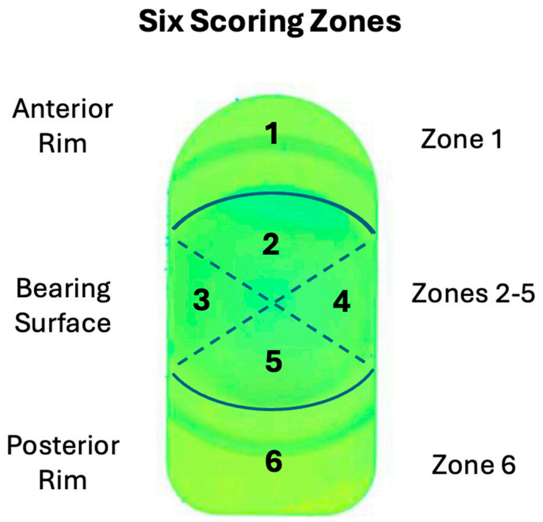

Figure S1. Diagram of L-TJR component damage-scoring regions for validation activities 1 and 2.

Validation Goal 1

Qualitatively, the lateral bending, axial rotation, and plasticity of the component resulted in predominantly asymmetric contact stress contours across the duty cycle (**Figure S2**, top). In general, contact pressure contours generally depicted loading across the entire area of the bearing surface depending on the phase of the duty cycle. The peak contact pressure was 33.6 MPa at or near to 50% of the duty cycle.

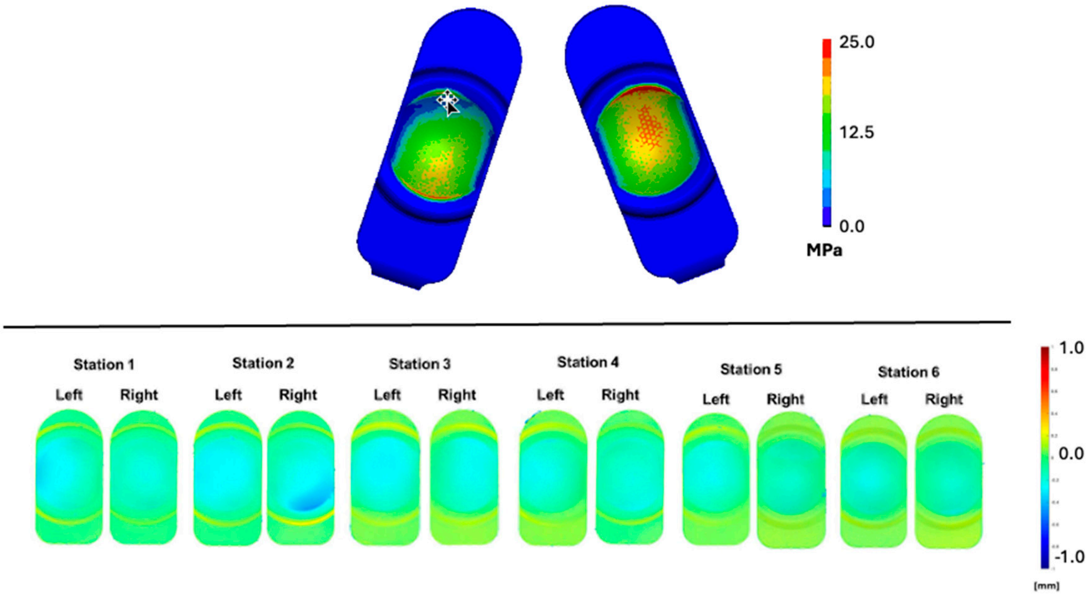

Figure S2: Contour plots representing the superposition of maximum contact pressures over the duty cycle. Superior component penetration maps based on microCT scans after 10 million cycles of Mode I duty cycles are also shown (bottom).

The 10 million cycle (MC) wear maps shown in **Figure S2** (bottom) for stations 1 to 6 were damage-scored for penetration, with fringes of the lightest blue corresponding to a deformation of greater than 0.2 mm and to visually differentiable damage from the wear map fringe plots. Similarly, the lightest blue fringes in the cumulative contact stress contour plots (>2.5 MPa) were judged to be associated with damage in the PE FEM fringe plot (**Figure S2, top**). The average total damage score for the penetration maps was 11.3 and was, on average, 12, for the FEM stress plots, corresponding to a difference of 6.2%. Overall, both the qualitative assessment as well as the semi-quantitative comparison of the simulator penetration maps and the cumulative contact stress results supported the credibility of the FEM under Mode I conditions.

Validation Goal 2

Qualitatively, the physical Mode IV wear testing indicated focal wear and deformation on the posterior aspect of the PE component, outside of the intended bearing surface as anticipated. These data were generally consistent with the standalone FEA results. In general, wear scar locational results between the short and long implants were similar (**Figures S3 and S4**). The average total damage scores for the penetration maps were 7.5 and 8.8 for the long and short designs, respectively. For the FEM stress plots, the average damage scores were 7 and 9 for the long and short designs, respectively. These differences corresponded to a difference of 6.7% and 2.2%. Overall, this credibility activity supported the validation of the FEM under Mode IV conditions.

The peak contact pressure for the long implant was 83.3 MPa and 104.8 MPa for the short. The peak von Mises stresses were 32.2 MPa and 41.8 MPa and the peak effective strains were 42% and 64% for the long and short implants, respectively. These values provide an upper-bound benchmark for the subsequent misalignment testing.

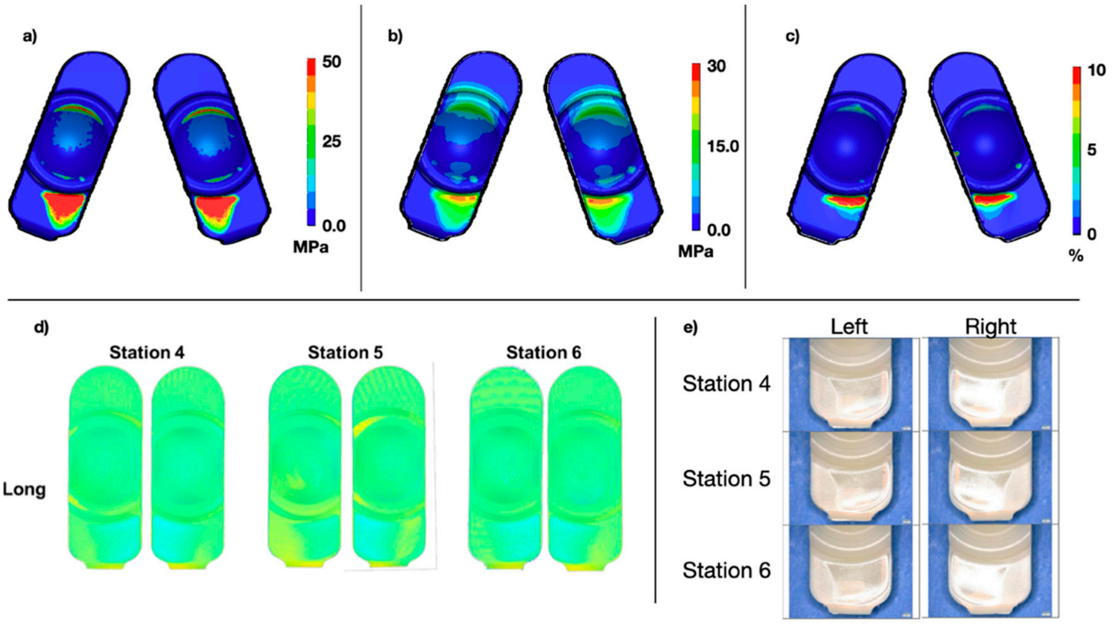

Figure S3. Contours of maximum contact pressure across all states (a), maximum von Mises stress across all states (b), and superposition of all plastic strains over the duty cycle (c) for the long design under Mode IV conditions. Superior component penetration maps based on microCT scans (d) and photo documentation (e) after 1 million cycles of Mode IV duty cycles are also shown.

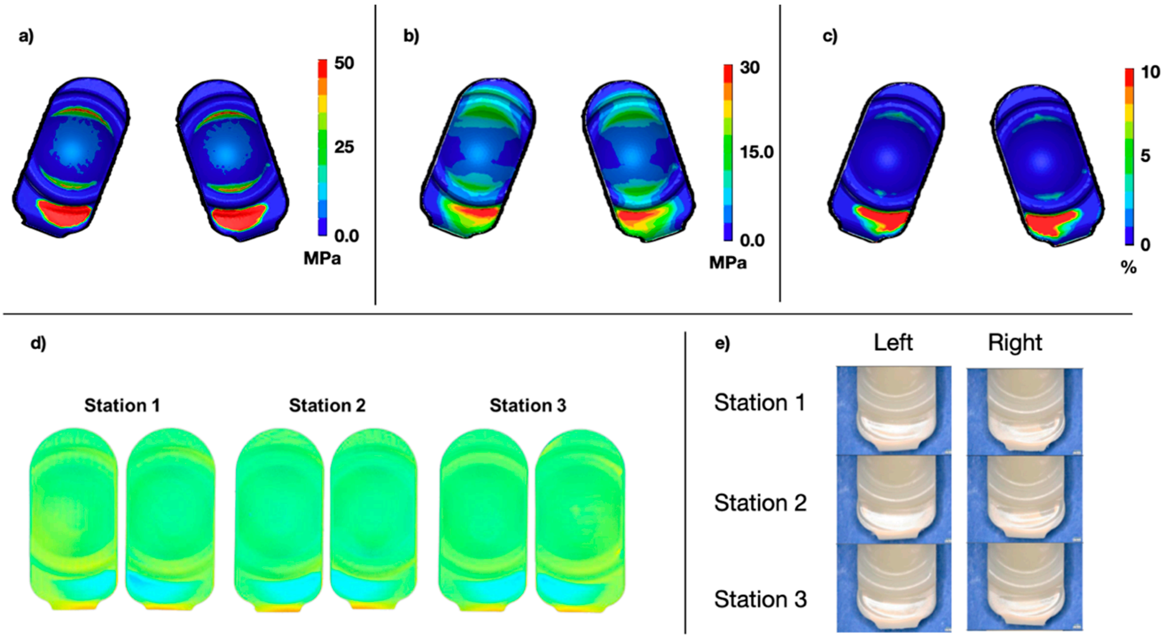

Figure S4. Contours of maximum contact pressure across all states (a), maximum von Mises stress across all states (b), and superposition of all plastic strains over the duty cycle (c) for the short design under Mode IV conditions. Superior component penetration maps based on microCT scans (d) and photo documentation (e) after 1 million cycles of Mode IV duty cycles are also shown.

<u>Validation Goal 3</u>

Bartel et al., 1995 [16] reported max contact pressures ranging from 40 to 60 MPa, which is generally greater than what was reported in the current model (~37 MPa). Given the greater axial load (3,000 N) and the generally stiffer PE used by Bartel et al., these results are consistent with those determined in the L-TJR FEM.

In a study by Rawlinson et al., 2006 [39], peak axial compressive loading of 2,000 N was applied to an FEM of a total knee replacement PE bearing surface. The PE material properties were the same as those described in Bartel et al., 1995. The authors reported maximum contact pressures of 26 and 30 MPa for two different TKR designs. These results are more similar to the results of the current FEM.

<u>Summary of Credibility Activities for FEM</u>

Overall, the FEA results were consistent with physical Mode I and IV penetration maps, as well as relevant published studies (Validation Goals 1-3). Thus, the results of these verification and validation activities support the conclusion that the L-TJR FEM is credible for its context of use.

Supplementary Material 2: FEM Results

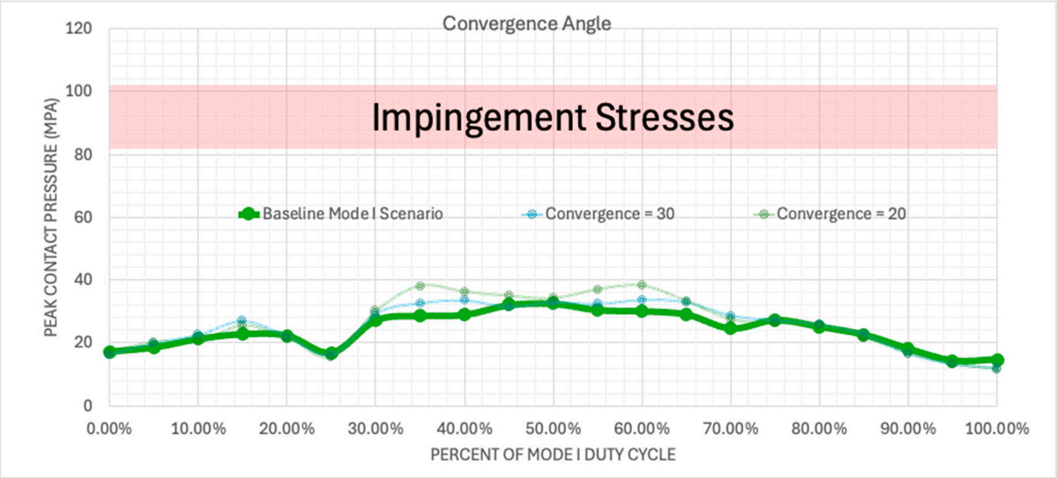

Figure S5. Plotted peak contact stress values for the Mode I duty cycle for the baseline, 30°, and 20° convergence angle simulations.

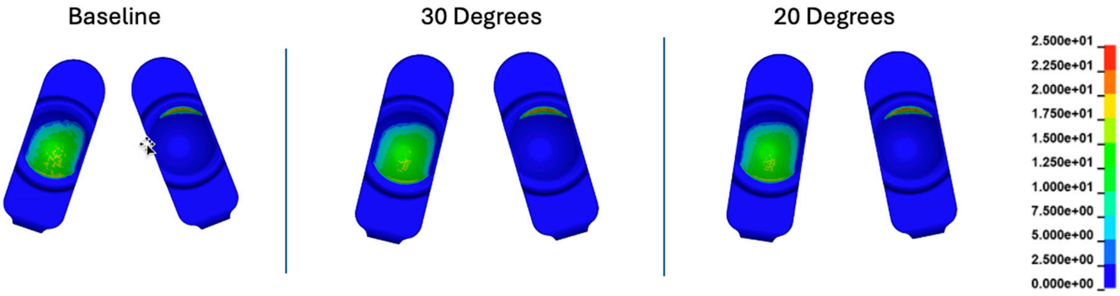

Figure S6. Contour plots of contact stress (MPa) at 35% of the Mode I duty cycle for the baseline, 30 degree, and 20 degree convergence angle simulations.

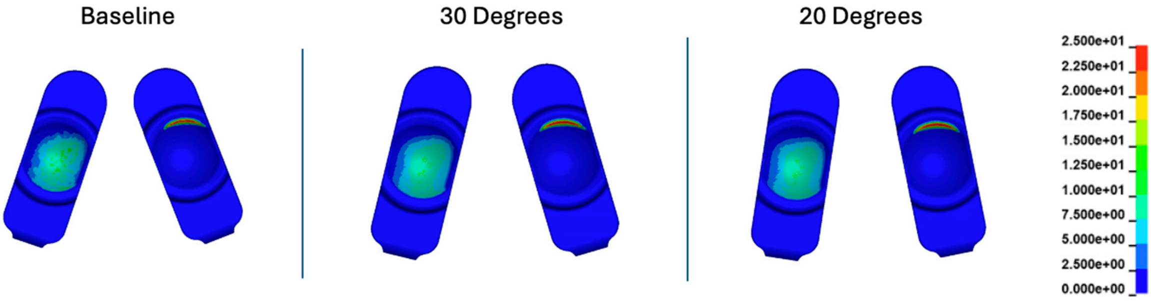

Figure S7. Contour plots of contact stress (MPa) at 60% of the Mode I duty cycle for the baseline, 30 degree, and 20 degree convergence angle simulations.

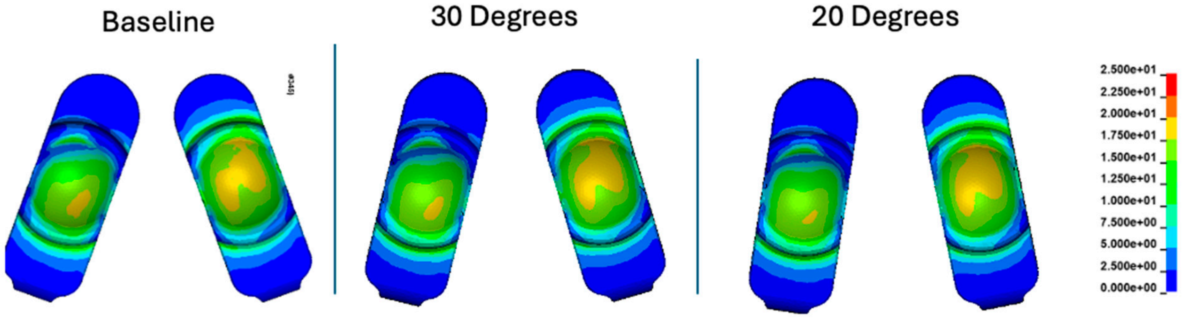

Figure S8. Contour plots representing the superposition of all von Mises stresses (MPa) over the duty cycle for the baseline, 30°, and 20° convergence angle simulations.

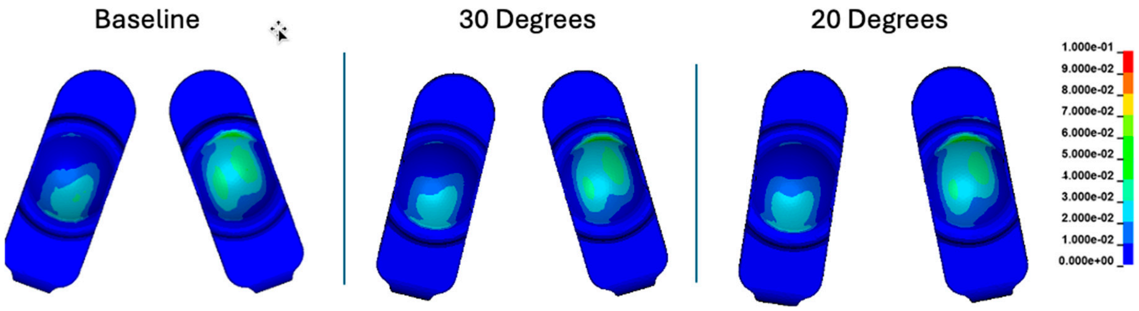

Figure S9. Contour plots representing the superposition of all effective strains over the duty cycle for the baseline, 30°, and 20° convergence angle simulations.

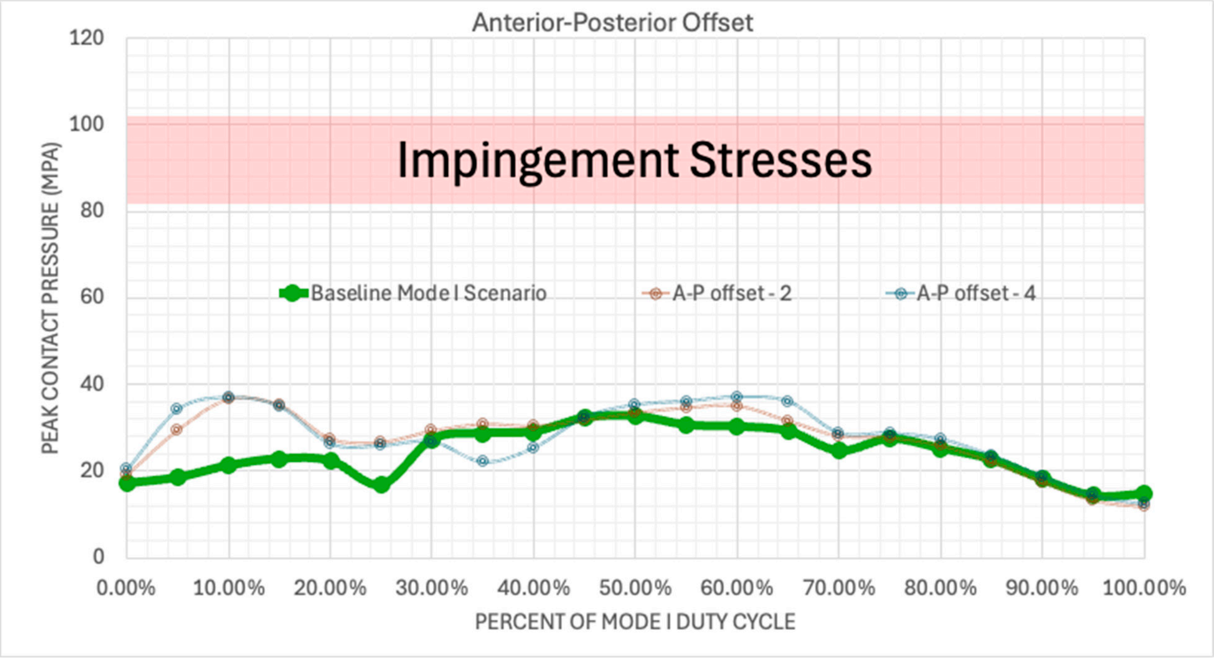

Figure S10. Plotted peak contact pressure values for the Mode I duty cycle for the 2 mm A-P offset, and 4 mm A-P offset simulations.

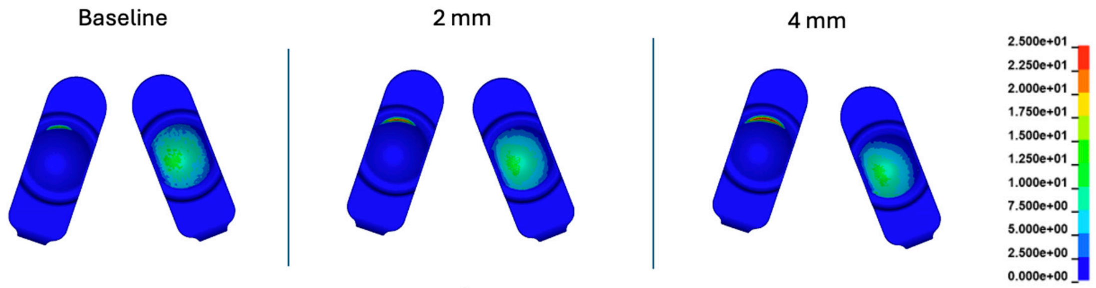

Figure S11. Contour plots of contact pressure at 10% of the Mode I duty cycle for the 2 mm A-P offset, and 4 mm A-P offset simulations.

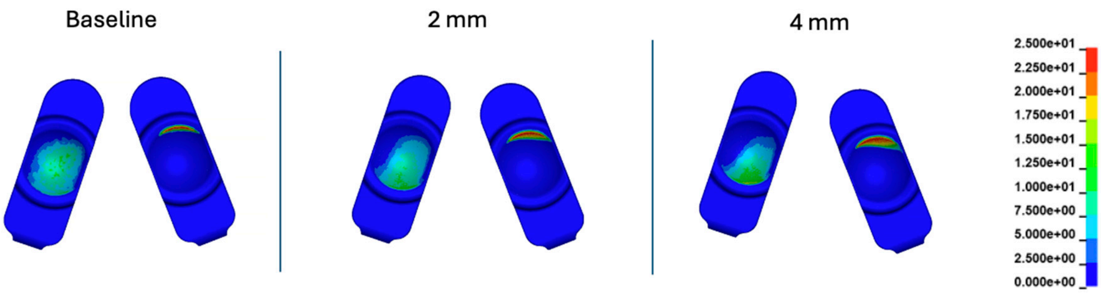

Figure S12. Contour plots of contact pressure at 60% of the Mode I duty cycle for the 2 mm A-P offset, and 4 mm A-P offset simulations.

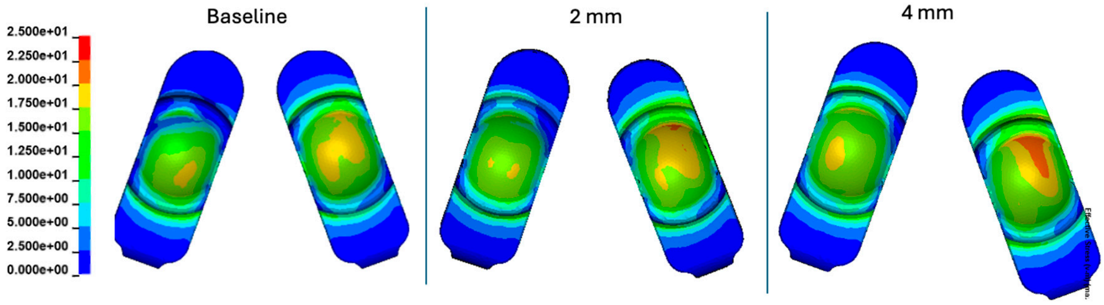

Figure S13. Contour plots representing the superposition of all von Mises stresses over the duty cycle for the 2 mm A-P offset, and 4 mm A-P offset simulations.

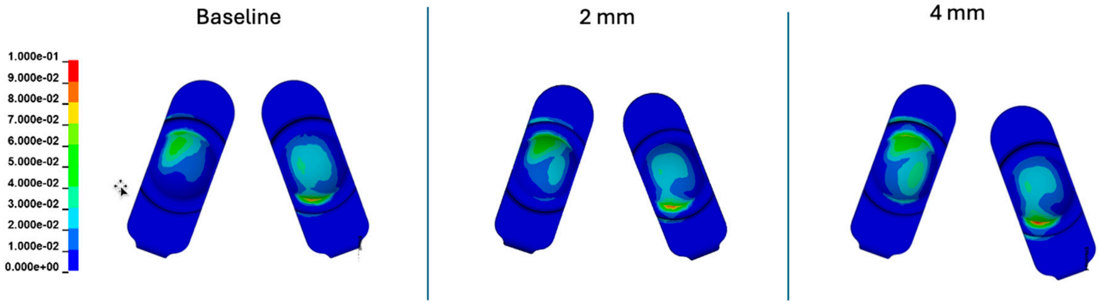

Figure S14. Contour plots representing the superposition of all effective strains over the duty cycle for the 2 mm A-P offset, and 4 mm A-P offset simulations.

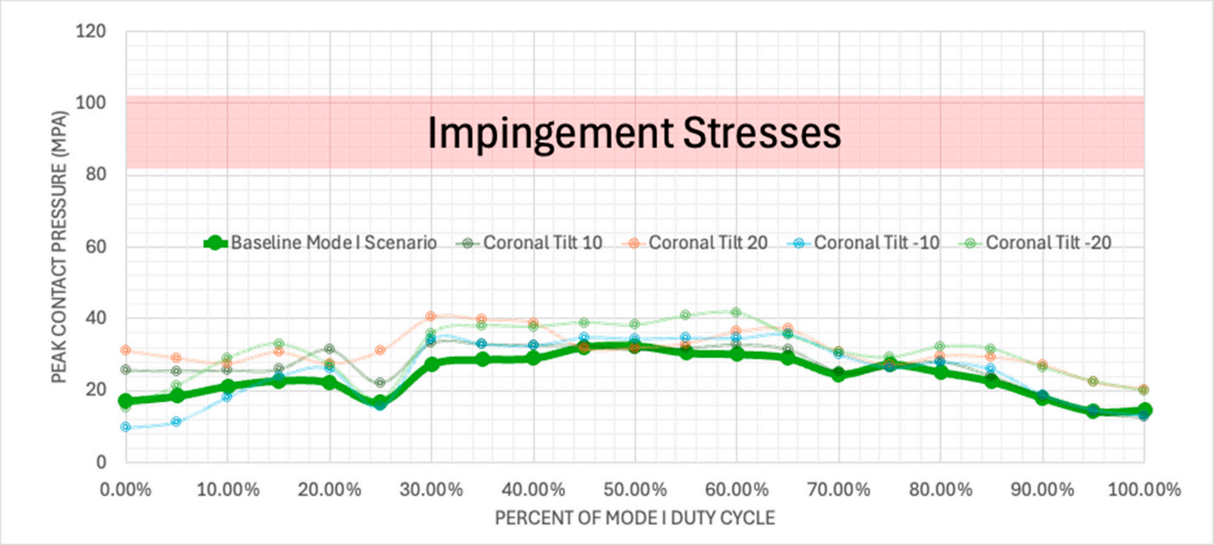

Figure S15. Plotted peak contact stress values for the Mode I duty cycle for the baseline, -20°, -10°, 10°, and 20° of coronal tilt simulations.

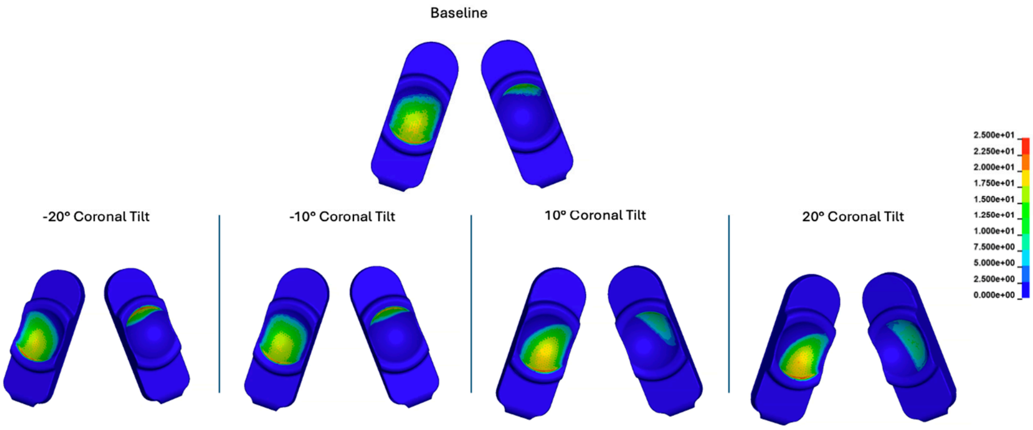

Figure S16. Contour plots of contact stress at 30% of the Mode I duty cycle for the baseline, -20 degrees, -10 degrees, 10 degrees, and 20 degrees of coronal tilt simulations.

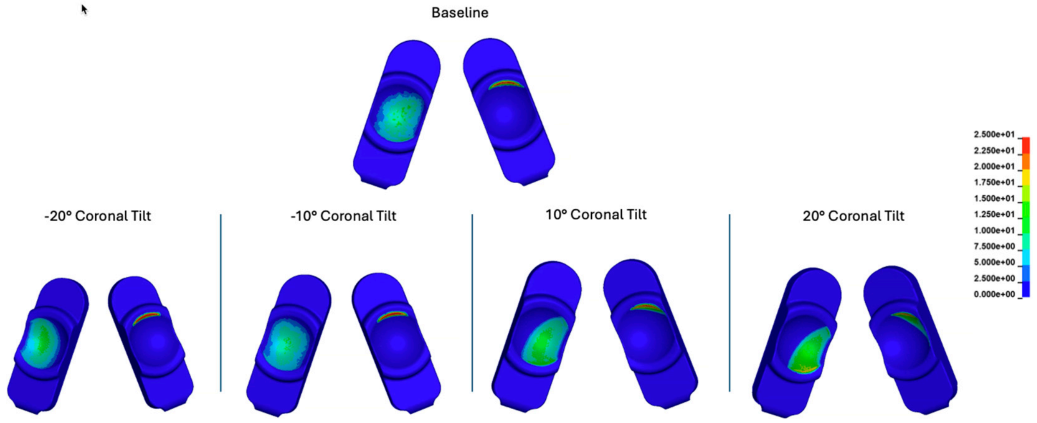

Figure S17. Contour plots of contact stress at 60% of the Mode I duty cycle for the baseline, -20 degrees, -10 degrees, 10 degrees, and 20 degrees of coronal tilt simulations.

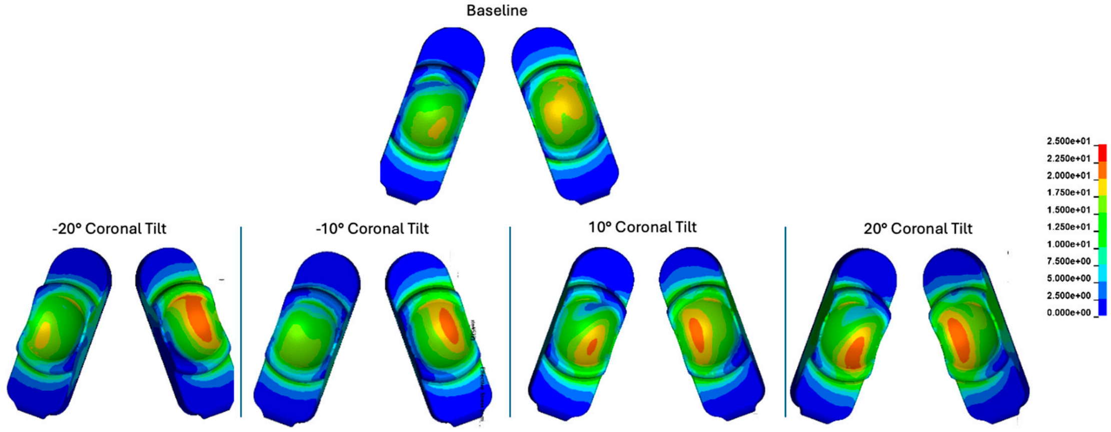

Figure S18. Contour plots representing the superposition of all von Mises stresses over the duty cycle for the baseline, -20°, -10°, 10°, and 20° of coronal tilt simulations.

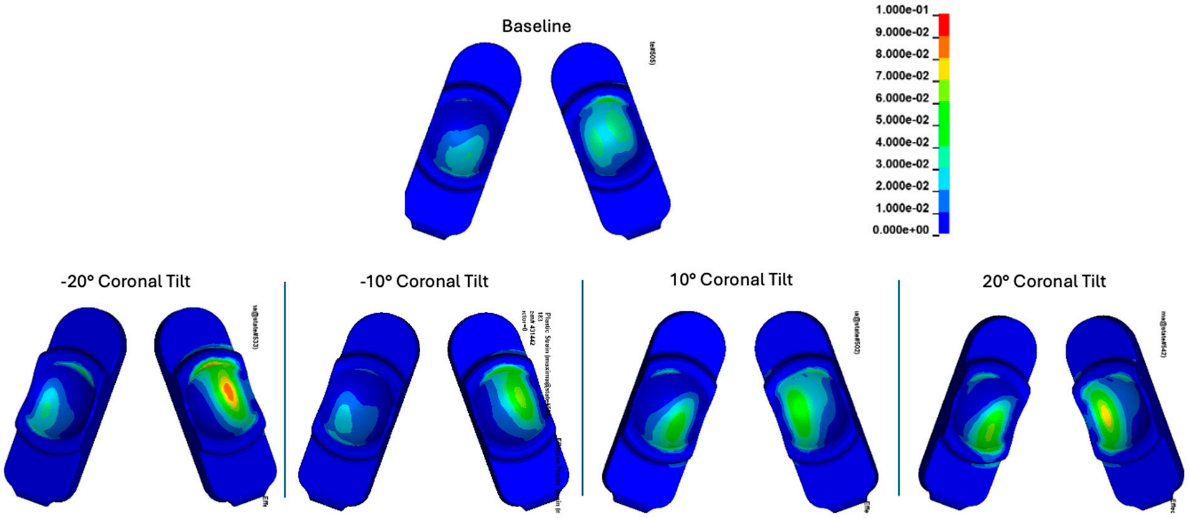

Figure S19. Contour plots representing the superposition of all effective strains over the duty cycle for the baseline, -20°, -10°, 10°, and 20° of coronal tilt simulations.
